# Sirt1-deficiency causes defective protein quality control

**DOI:** 10.1038/srep12613

**Published:** 2015-07-29

**Authors:** Takuya Tomita, Jun Hamazaki, Shoshiro Hirayama, Michael W. McBurney, Hideki Yashiroda, Shigeo Murata

**Affiliations:** 1 Laboratory of Protein Metabolism, Graduate School of Pharmaceutical Sciences, The University of Tokyo, Tokyo, Japan; 2 Center for Cancer Therapeutics, Ottawa Hospital Research Institute, Ottawa, Ontario, Canada

## Abstract

Protein quality control is an important mechanism to maintain cellular homeostasis. Damaged proteins have to be restored or eliminated by degradation, which is mainly achieved by molecular chaperones and the ubiquitin-proteasome system. The NAD^+^-dependent deacetylase Sirt1 has been reported to play positive roles in the regulation of cellular homeostasis in response to various stresses. However, its contribution to protein quality control remains unexplored. Here we show that Sirt1 is involved in protein quality control in both an Hsp70-dependent and an Hsp70-independent manner. Loss of Sirt1 led to the accumulation of ubiquitinated proteins in cells and tissues, especially upon heat stress, without affecting proteasome activities. This was partly due to decreased basal expression of Hsp70. However, this accumulation was only partially alleviated by overexpression of Hsp70 or induction of Hsp70 upon heat shock in Sirt1-deficient cells and tissues. These results suggest that Sirt1 mediates both Hsp70-dependent and Hsp70-independent protein quality control. Our findings cast new light on understanding the role of Sirt1 in maintaining cellular homeostasis.

A critical component of cellular homeostasis is protein quality control, which maintains the integrity of the proteome. Proteins often misfold under stress conditions or as a result of genetic mutations or translation errors. Such misfolded proteins can be deleterious to cells, as evidenced by many neurodegenerative diseases such as Alzheimer’s disease, Huntington’s disease, amyotrophic lateral sclerosis, and Parkinson’s disease, in which aggregation of tau, polyglutamine proteins, SOD1, and α-synuclein are observed, respectively^1^,^2^.

Molecular chaperones and the ubiquitin-proteasome system play pivotal roles in protein quality control^3^. Molecular chaperones assist protein folding and prevent protein misfolding, especially against cellular stresses that cause denaturation of proteins^4^. Proteins that fail to attain a correct conformation are recognized by the ubiquitin system, ubiquitinated, and mainly degraded by the proteasome^5^,^6^. Molecular chaperones such as Hsp70 are also shown to have a contribution to ubiquitination of unfolded and misfolded proteins^7^,^8^. Impairment of protein quality control causes accumulation of aberrant proteins and affects the integrity and longevity of organisms^9^.

Sirtuins are NAD^+^-dependent deacetylases that play important roles in maintaining cellular homeostasis. Although classified as class III histone deacetylases, they act not only on histones but also on many other proteins that play key roles in various cellular signaling pathways^10^. Mammals possess seven sirtuins, Sirt1–7, of which Sirt1 is best characterized. Sirt1 responds to various intracellular stresses such as caloric restriction, oxidative stress, and DNA damage^11^. The enzymatic activity of Sirt1 is also associated with age-related diseases such as cancer, Alzheimer’s disease, and type II diabetes as well as longevity in mouse models^12^,^13^. Recent reports have implicated a relationship between Sirt1 and protein quality control. Sirt1 suppresses aggregation of tau proteins by directly deacetylating them^14^. Sirt1 activity in p25-overexpression mice, a model of Alzheimer’s disease and tauopathies, was shown to protect against neurodegeneration^15^. Sirt1 also positively regulates autophagy, which is another bulk degradation process in eukaryotic cells^16^,^17^. In addition, Sirt1 deacetylates heat shock transcription factor 1 (HSF1) and augments binding of HSF1 to its cognate promoter^18^,^19^. Therefore, it is possible that Sirt1 is involved in protein quality control in various ways. However, it remains unclear whether Sirt1 is involved in quality control mediated by the ubiquitin-proteasome system.

In this study, we studied how Sirt1-deficiency affects protein quality control with respect to ubiquitin-dependent protein degradation and found that Sirt1-deficiency led to accumulation of ubiquitinated proteins in cells without affecting proteasome activities. Sirt1-deficiency decreased basal expression of Hsp70 and showed defects in Hsp70-mediated ubiquitin-dependent protein degradation. However, this accumulation of ubiquitinated proteins was only partially alleviated by overexpression of Hsp70 in Sirt1^–/–^ cells and tissues. These results suggest that Sirt1 mediates both Hsp70-dependent and Hsp70-independent protein quality control.

## Results and Discussion

### Ubiquitinated proteins are increased in Sirt1-deficient MEFs

**Figure 1:**
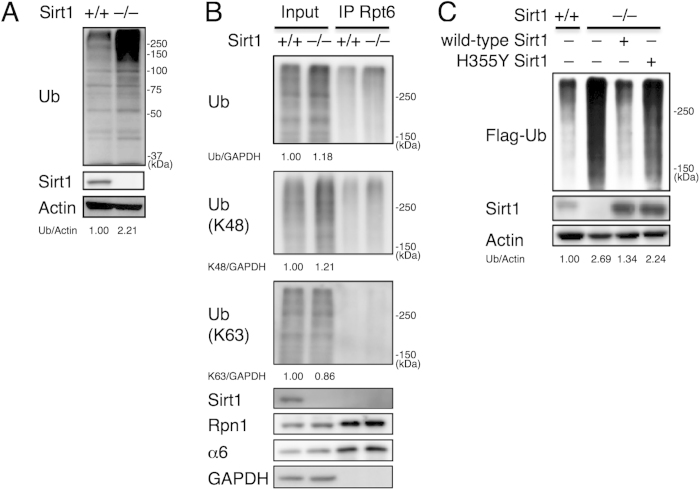
Ubiquitinated proteins accumulate in Sirt1^–/–^ MEFs. (**A**) Immunoblotting of immortalized MEF lysates of the indicated genotype. Actin serves as a loading control. Values represent the relative band intensities of ubiquitin (Ub) (normalized to GAPDH). (**B**) Lysates from wild-type and Sirt1^–/–^ MEFs were subjected to immunoprecipitation with anti-Rpt6 antibodies, followed by immunoblotting. Values represent the relative band intensities of Ub (normalized to GAPDH). (**C**) Immunoblotting of lysates from Flag-ubiquitin-introduced wild-type MEFs and Sirt1^–/–^ MEFs transfected with wild-type Sirt1 or H355Y Sirt1. Values represent the relative band intensities of Ub (normalized to Actin). Uncropped gel images are shown in Supplementary Figure.

To examine the role of Sirt1 in protein quality control, we used immortalized mouse embryonic fibroblasts derived from Sirt1-deficient mice (Sirt1^–/–^ MEFs)^20^. When the lysates from Sirt1^–/–^ and wild-type MEFs were subjected to Western blotting, we found that ubiquitinated proteins were markedly increased in Sirt1^–/–^ MEFs (Fig. 1A).

To address the physiological significance of the increase in ubiquitinated proteins, it is important to define the linkage type of poly-ubiquitin chains. Ubiquitin has seven lysine residues (K6, K11, K27, K29, K33, K48, and K63) and an N-terminal methionine (M1) that can form poly-ubiquitin chains. For instance, K48-linked poly-ubiquitin chains are known to serve as a signal of protein degradation by the proteasome, while K63-linked chains are involved in non-proteolytic function such as DNA damage response and NF-κB signaling^21^. Antibodies specific to either K48- or K63-linked ubiquitin chains revealed that increased ubiquitinated proteins in Sirt1^–/–^ MEFs were conjugated with K48-linked ubiquitin chains and not K63-linked chains, suggesting that the increased ubiquitinated proteins were the substrates of the proteasomes (Fig. 1B, lanes 1 and 2). Consistent with this view, when the lysates were immunoprecipitated with an antibody against the proteasome subunit Rpt6, a larger amount of K48-linked ubiquitinated proteins was pulled down in Sirt1^–/–^ MEFs than wild-type MEFs, while the amount of two other coprecipitated proteasome subunits, Rpn1 and α6, were comparable to each other (Fig. 1B). We also observed that Sirt1 did not coprecipitate with the proteasome, suggesting that Sirt1 is not constitutively associated with the proteasome (Fig. 1B).

To exclude the possibility that the increase in ubiquitinated proteins was simply due to increased expression of endogenous ubiquitin, we transfected Flag-tagged ubiquitin exogenously in wild-type and Sirt1^–/–^ MEFs so that each cell line expressed an equal amount of Flag-ubiquitin, and observed the amount of Flag-ubiquitin-conjugated proteins. This again showed that Sirt1^–/–^ MEFs accumulated ubiquitinated proteins compared to wild-type MEFs (Fig. 1C, lanes 1 and 2). To examine whether this accumulation was dependent on the deacetylase activity of Sirt1, wild-type or a catalytically inactive H355Y mutant Sirt1 was expressed in Sirt1^–/–^ MEFs. While introduction of wild-type Sirt1 reduced the amount of ubiquitinated proteins to a level similar to wild-type cells, H355Y Sirt1 did not (Fig. 1C, lanes 3 and 4). These results indicate that the deacetylase activity of Sirt1 is important for preventing accumulation of ubiquitinated proteins in cells.

### Proteasome activities are not impaired in Sirt1-deficient MEFs

**Figure 2:**
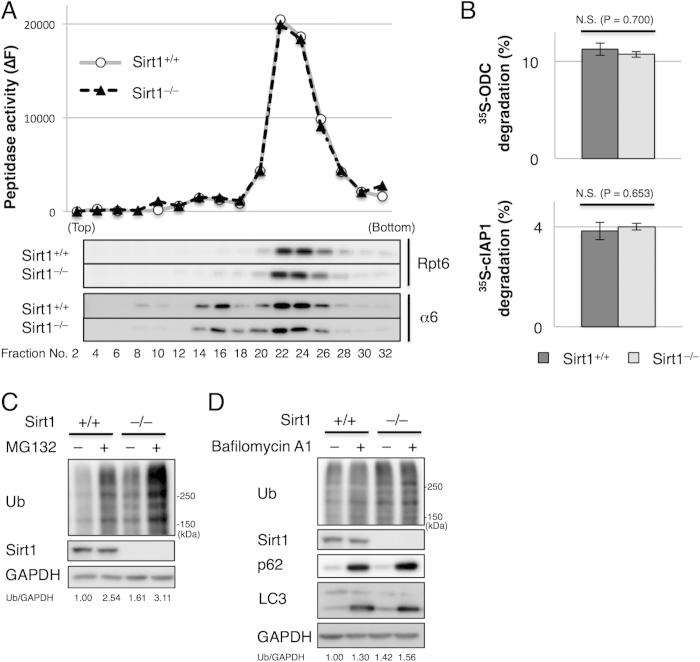
Proteasome activities are not impaired in Sirt1^–/–^ MEFs. (**A**) Lysates from wild-type and Sirt1^–/–^ MEFs were fractionated by 8–32% glycerol gradient centrifugation. An aliquot of each fraction was used for an assay of chymotryptic activity of proteasomes using succinyl-Leu-Leu-Val-Tyr-7-amino-4-methyl-coumarin (Suc-LLVY-AMC) as a substrate and immunoblotting for Rpt6 and α6. (**B**) Ubiquitin-independent and -dependent protease activities of proteasomes. Lysates from wild-type and Sirt1^–/–^ MEFs were subjected to an *in vitro* protein degradation assay. Antizyme-dependent degradation of ^35^S-labeled ODC and ubiquitin-dependent degradation of ^35^S-labeled cIAP1 were measured. The data represent means ± standard error of the mean (SEM) from three independent experiments. Statistical comparisons were made by Student’s *t*-test for two tailed unpaired samples. N.S. indicates not significant. (**C**) Lysates of wild-type and Sirt1^–/–^ MEFs treated with or without 10 μM MG132 for 30 min were subjected to immunoblotting with the indicated antibodies. Values represent the relative band intensities of Ub (normalized to GAPDH). (**D**) Lysates of wild-type and Sirt1^–/–^ MEFs treated with or without 100 nM bafilomycin A1 for 20 h were subjected to immunoblotting with the indicated antibodies. Values represent the relative band intensities of Ub (normalized to GAPDH). Uncropped gel images are shown in Supplementary Figure.

Both overproduction of ubiquitinated proteins and a decrease in the degradation of ubiquitinated proteins by the proteasome or autophagy can lead to an increase in ubiquitinated proteins in cells. We first examined whether proteasome activity was compromised in the absence of Sirt1. The lysates of wild-type and Sirt1^–/–^ MEFs were fractionated by glycerol gradient centrifugation, followed by the measurement of the peptidase activity of the proteasome and immunoblot analysis of each fraction. The peptidase activities were almost identical between wild-type and Sirt1^–/–^ MEFs (Fig. 2A, upper panel). Also, immunoblot analysis of the proteasome subunits Rpt6 and α6 did not show any differences between the two cell lines, indicating that the amount of the proteasome is not affected by lack of Sirt1 (Fig. 2A, lower panels).

To assess proteasome activity in the context of degradation of native proteins, we measured the *in vitro* degradation rate of two substrates that are degraded by the proteasome in an ATP-dependent manner. One is ornithine decarboxylase, which is degraded in a ubiquitin-independent and an antizyme-dependent manner^22^. The other is inhibitor of apoptosis-1 (cIAP1), which is degraded in a ubiquitin-dependent manner^23^. However, no differences were observed in the degradation of these substrates between wild-type and Sirt1^–/–^ MEFs (Fig. 2B). Taken together, these results suggest that proteasome function in Sirt1^–/–^ MEFs is normal. Consistent with this, a marked increase in ubiquitinated proteins was observed both in wild-type and Sirt1^–/–^ MEFs when cells were treated with the proteasome inhibitor MG132 (Fig. 2C).

Previous studies reported that Sirt1 positively regulates autophagy^16^,^17^. Therefore, we next examined whether accumulation of ubiquitinated proteins in Sirt1^–/–^ MEFs is due to defective autophagy. Accumulation of the autophagy-specific substrate p62 was not observed in Sirt1^–/–^ MEFs (Fig. 2D), indicating that autophagy is not impaired in Sirt1^–/–^ MEFs. Inhibition of lysosomal degradation by bafilomycin A1 resulted in a marked increase in p62 and LC3-II and a modest increase in ubiquitin conjugates both in wild-type and Sirt1^–/–^ MEFs (Fig. 2D). These results indicate that autophagy is active in Sirt1^–/–^ MEFs and suggests that autophagy impairment is not the primary cause of accumulation of ubiquitinated proteins in Sirt1^–/–^ MEFs.

Taken together, these results suggest that increased ubiquitinated proteins in Sirt1^–/–^ MEFs were not a result from decreased degradation either by the proteasome or autophagy. Rather, it is likely that ubiquitination of some proteins is increased in Sirt1^–/–^ MEFs.

### Sirt1-deficient MEFs are defective in Hsp70-dependent protein quality control

**Figure 3:**
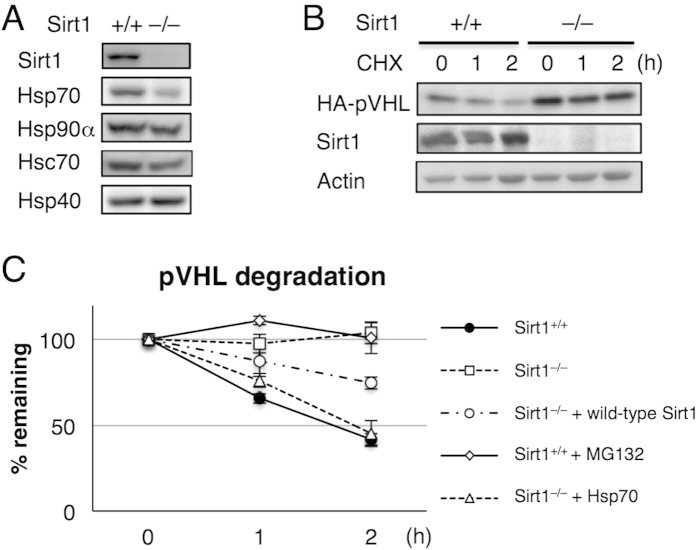
Hsp70-dependent protein quality control is impaired in Sirt1^–/–^ MEFs. (**A**) Immunoblotting of lysates from wild-type and Sirt1^–/–^ MEFs. (**B**) Degradation of pVHL was monitored by immunoblotting. HA-tagged pVHL was transfected into wild-type and Sirt1^–/–^ MEFs, and 100 μg/ml cycloheximide (CHX) was added prior to harvest at the indicated times. Actin serves as a loading control. (**C**) Cycloheximide chase as described in (**B**) was performed in wild-type, Sirt1^–/–^, Sirt1^–/–^ transfected with wild-type Sirt1 or Hsp70, and wild-type MEFs treated with 10 μM MG132. Protein levels of HA-pVHL were quantified. Actin was used for normalization. The data represent means ± SEM from three independent experiments. Uncropped gel images are shown in Supplementary Figure.

Since the proteasome and autophagy were not impaired in Sirt1^–/–^ MEFs, we next examined whether Sirt1-deficiency increased ubiquitination of proteins. It has been previously reported that Sirt1-mediated deacetylation of heat shock transcription factor 1 (HSF1) promotes HSF1 binding to the heat shock promoter^18^,^19^. Accordingly, we examined the expression levels of four representative heat shock proteins Hsp90α, Hsc70, Hsp70, and Hsp40 in wild-type and Sirt1^–/–^ MEFs. This revealed that Hsp70 was remarkably decreased in Sirt1^–/–^ MEFs while the others remained unchanged (Fig. 3A).

We next examined the degradation rate of von Hippel-Lindau protein (pVHL), which was reported to be a short-lived protein and require Hsp70 to be degraded by the ubiquitin-proteasome pathway^24^. In wild-type MEFs, transfected HA-tagged pVHL was rapidly degraded, which was inhibited by the proteasome inhibitor MG132 (Fig. 3B,C). In contrast, Sirt1^–/–^ MEFs showed significant delay in the degradation of HA-pVHL (Fig. 3B,C). This delay was rescued by expressing Sirt1 or Hsp70 (Fig. 3C). These results indicate that Sirt1-deficiency leads to a defect in proteasome-dependent degradation of pVHL and suggests that Hsp70-dependent protein degradation is impaired in the absence of Sirt1.

### Sirt1-deficiency affects protein quality control independent of Hsp70 expression

**Figure 4:**
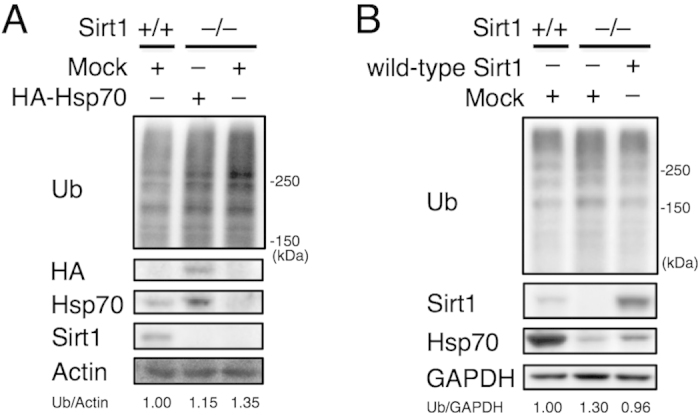
Downregulation of Hsp70 is not the sole cause of aberrant protein quality control in Sirt1^–/–^ MEFs. (**A**) Immunoblotting of lysates from wild-type and Sirt1^–/–^ MEFs transfected with empty vector or HA-tagged Hsp70. Values represent the relative band intensities of Ub (normalized to Actin). (**B**) Immunoblotting of lysates from wild-type, Sirt1^–/–^, and Sirt1^–/–^ transfected with wild-type Sirt1 MEFs. Values represent the relative band intensities of Ub (normalized to GAPDH). Uncropped gel images are shown in Supplementary Figure.

We next asked whether decreased expression of Hsp70 is the cause of accumulation of ubiquitinated proteins in Sirt1^–/–^ MEFs, as proteins that fail to fold correctly are usually ubiquitinated for proteasomal degradation^5^. We expressed Hsp70 in Sirt1^–/–^ MEFs. Compared to Sirt1^–/–^ MEFs without Hsp70 transfection, the amount of ubiquitinated proteins was slightly reduced in Sirt1^–/–^ MEFs overexpressing Hsp70, but not to the extent of wild-type MEFs (Fig. 4A). These results suggest that decreased expression of Hsp70 is involved in the accumulation of ubiquitinated proteins in the absence of Sirt1, but is not the sole cause. This view was further supported when wild-type Sirt1 was overexpressed in Sirt1^–/–^ MEFs. While recovery of Hsp70 was only partial in Sirt1^–/–^ MEFs transfected with Sirt1, the amount of ubiquitinated proteins was reduced to a level comparable to wild-type MEFs (Fig. 4B). These results suggest that Sirt1 plays an important role in protein quality control in part by promoting expression of Hsp70 but also by a mechanism independent of Hsp70.

### Sirt1 is not required for the expression of Hsp70 during post-heat shock recovery

**Figure 5:**
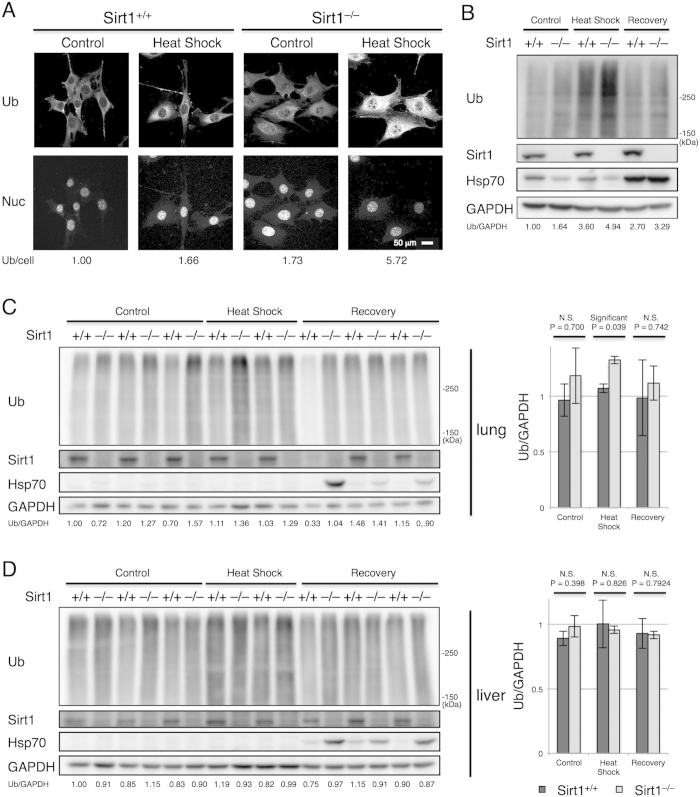
Sirt1-deficiency does not lead to impairment in the induction of Hsp70 after heat shock. (**A**) Immunofluorescence with anti-ubiquitin antibodies of wild-type and Sirt1^–/–^ MEFs treated with or without heat shock (42 °C for 30 min). Nuclei were stained with Hoechst 33342. Scale bar, 50 μm. Values represent the relative intensity of Ub staining per cell. (**B**) Wild-type and Sirt1^–/–^ MEFs were treated with or without heat shock (42 °C for 30 min). Heated cells were then immediately harvested or allowed to recover at 37 °C for 5 h, and cell lysates were subjected to immunoblotting. Values represent the relative band intensities of Ub (normalized to GAPDH). (**C**,**D**) Wild-type and Sirt1^–/–^ mice were treated with or without heat shock (42 °C for 45 min). Each group consisted of four or six littermates. Heated mice were then immediately sacrificed or allowed to recover at room temperature for 7 h. The lysates from their lung (**C**) and liver (**D**) were subjected to immunoblotting (left panels). Values represent the relative band intensities of Ub (normalized to GAPDH). Statistical comparisons were made by Student’s *t*-test for two tailed unpaired samples. The data represent means ± SEM. N.S. indicates not significant. Uncropped gel images are shown in Supplementary Figure.

We then examined whether proteins are vulnerable to proteotoxic stresses in the absence of Sirt1. When wild-type and Sirt1^–/–^ MEFs were exposed to heat shock, marked accumulation of ubiquitinated proteins in Sirt1^–/–^ MEFs was observed, compared to wild-type MEFs (Fig. 5A). The increase of ubiquitinated proteins in Sirt1^–/–^ MEFs was confirmed by Western blotting after heat shock. Intriguingly, Hsp70 was strongly induced not only in wild-type MEFs but also in Sirt1^–/–^ MEFs during the recovery phase after heat shock (Fig. 5B). Nevertheless, the amount of ubiquitinated proteins was still higher in Sirt1^–/–^ MEFs than wild-type MEFs (Fig. 5B). These results indicate that a larger amount of proteins are subjected to ubiquitination after heat shock in the absence of Sirt1 and again suggest that Sirt1 exerts Hsp70-independent protein quality control. These results also raise a question about the regulation of HSF1 by Sirt1. Although it has been suggested that deacetylation of HSF1 by Sirt1 after heat shock plays an important role in activation of HSF1 transcriptional activity^25^, our data indicates that Sirt1-deficiency does not lead to loss of heat shock response.

We further investigated the impact of Sirt1-deficiency in mouse tissues with regard to the amount of ubiquitinated proteins and heat shock response. While basal expression of Hsp70 was comparable between wild-type and Sirt1^–/–^ in the lung, Sirt1^–/–^ lungs had significantly increased levels of ubiquitinated proteins, especially after heat shock (Fig. 5C). Intriguingly, strong induction of Hsp70 was observed in Sirt1^–/–^ lungs but not in wild-type lungs during the recovery phase (Fig. 5C). Stronger induction of Hsp70 in the absence of Sirt1 was also observed in the liver, although the increase in ubiquitinated proteins in Sirt1^–/–^ livers was not as apparent as in Sirt1^–/–^ lungs (Fig. 5D). It is unclear why Hsp70 was more intensely induced in the absence of Sirt1, but it may be possible that an alternative pathway to induce Hsp70 other than deacetylated HSF1 is sensitized in Sirt1^–/–^ mice because of the underlying defect in protein quality control. The observation that Sirt1^–/–^ lungs are susceptible to accumulation of ubiquitinated proteins compared to the liver might be relevant to the phenotype of Sirt1^–/–^ mice, where defects in the lung, but not in the liver, were reported previously^20^.

In sum, we showed that Sirt1 is involved in protein quality control both in an Hsp70-dependent and independent manner. Overexpression of Hsp70 in Sirt1^–/–^ MEFs partially decreased ubiquitinated proteins and promoted degradation of pVHL, which requires Hsp70 (Fig. 3B,C). However, the effect is not to the extent observed in wild-type MEFs (Fig. 4A). In addition, although the recovery of Hsp70 was only partial in Sirt1^–/–^ MEFs transfected with Sirt1, the amount of ubiquitinated proteins was reduced to a level comparable to wild-type MEFs (Fig. 4B). These results suggest that Hsp70 is not the sole factor of Sirt1-mediated protein quality control and that Sirt1 has a role other than inducing expression of Hsp70 in protein quality control, which may vary depending on cell types.

As reported previously, a large part of Sirt1-deficient mice died in the perinatal period and those that survived showed developmental defects^20^,^26^. Although it has been reported that Sirt1-deficiency affects the lung, pancreas, and heart, the molecular mechanisms remain elusive. Our findings suggest that protein quality control is an important role of Sirt1 and that a defect in protein quality control might be one of the causes of the phenotypes observed by Sirt1 deficiency.

## Methods

### MEF cells, transfection, and reagents

Immortalized Sirt1-decicient mouse embryonic fibroblast (MEF) cells and plasmids encoding mouse Sirt1 cDNAs (both wild-type and H355Y mutant) were described previously^20^. Transfections of plasmids into cells were performed using Lipofectamine 2000 (Invitrogen). To generate stable cell lines by retroviral infection, we used the pMXs vector series and 12 μg/ml blasticidin for selection^27^. MG132, bafilomycin A1, and cycloheximide were purchased from Peptide Instiute, Wako, and Sigma, respectively.

### Mice

Sirt1-deficient mouse strain (RBRC05323) was provided by RIKEN BRC through the National Bio-Resource Project of the MEXT, Japan. The mutant mice used in this study were 4 to 8 week-old and maintained on a mixed genetic background with the CD1 mouse strain under specific pathogen-free conditions. All animal protocols were approved by the Institutional Animal Care Committee of Graduate School of Pharmaceutical Sciences, the University of Tokyo (Approval #; M25-19) and carried out in accordance with the guidelines set by this committee.

### Biochemical assays

Cells were lysed with ice-cold phosphate-buffered saline (PBS) containing 0.5% Nonidet P-40 (NP-40). Mouse tissues were homogenized in RIPA buffer (50 mM Tris-HCl pH 7.5, 1 mM EDTA, 0.1% SDS, 150 mM NaCl, 1% NP-40, 0.1% sodium deoxycholate) including 1 mM PMSF. The extracts were clarified by centrifugation at 20,000 × g for 10 min at 4 °C and then boiled in SDS sample buffer in the presence of β-mercaptoethanol (β-ME). In heat shock response assays, cell culture dishses or mouse cages were submerged in a water bath kept at 42 °C for 30 or 45 min. For Western blotting, samples were subjected to SDS–PAGE, transferred to polyvinylidene fluoride membrane, and analyzed by immunoblotting. The gels have been run under the same experimental conditions. For immunofluorescence, cells were fixed with 4% paraformaldehyde and observed with TCS SP8 (Leica). The antibodies for Flag (Sigma, F1804), HA (MBL, 561), Sirt1 (Millipore, 07-131), Actin (Millipore, MAB1501R), GAPDH (Abcam, ab8245), ubiquitin (for immunoblotting of MEFs, Dako Cytomation, Z 0458), ubiquitin (for immunoblotting of tissues and for immunofluorescence, MBL, D058-3), K48-specific ubiquitin (Millipore, 05-1307), K63-specific ubiquitin (Millipore, 05-1308), p62 (MBL, PM045), LC3 (MBL, PM036), Hsp90α (MBL, SR-B971), Hsc70 (MBL, SR-B816), Hsp70 (MBL, SR-B812), and Hsp40 (Stressgen, SPA-400) were purchased. The antibodies for proteasome subunit α6, Rpn1, and Rpt6 were described previously^28^. Protein G for immunoprecipitation was purchased from GE Healthcare and Hoechst 33342 was from Nacalai. All images were taken with the same settings and processed exactly the same way. Quantification of bands was performed using Image Gauge software (for pVHL degradation assay, Fujifilm) and Fusion SL4 (for quantification of Ub, M&S Instruments).

### Glycerol gradient centrifugation analysis and assay of proteasome activity

Lysates of MEFs were clarified by centrifugation at 20,000 × g and subjected to 8 to 32% (vol/vol) linear glycerol gradient centrifugation (22 h, 83,000 × g) as described previously^29^. For immunoblot analysis, the fractionated samples were precipitated by cold acetone and dissolved in SDS sample buffer containing β-mercaptoethanol. The assays of proteasome chymotryptic peptidase activity, degradation of recombinant ^35^S-labeled ornithine decarboxylase (ODC), and degradation of polyubiquitinated ^35^S-labeled cIAP1 protein have been described previously^29^.

## Additional Information

**How to cite this article**: Tomita, T. *et al.* Sirt1-deficiency causes defective protein quality control. *Sci. Rep.* **5**, 12613; doi: 10.1038/srep12613 (2015).

## Supplementary Material

Supplementary Figure
