# Supplementary material for: Sirt1-deficiency causes defective protein quality control
Source: Sci Rep. 2015 Jul 29;5:12613. doi: 10.1038/srep12613 (PMC4518232; doi:10.1038/srep12613)
Supplement: Supplementary Figure [file srep12613-s1.pdf]

Supplementary Information

Sirt1-deficiency causes defective protein quality control

Takuya Tomita[1], Jun Hamazaki[1], Shoshiro Hirayama[1], Michael W. McBurney[2], Hideki Yashiroda[1] & Shigeo Murata[1*]

[1]Laboratory of Protein Metabolism, Graduate School of Pharmaceutical Sciences, The University of Tokyo, Tokyo, Japan
[2]Center for Cancer Therapeutics, Ottawa Hospital Research Institute, Ottawa, Ontario, Canada
[*]Correspondence and requests for materials should be addressed to S.M. (smurata@mol.f.u-tokyo.ac.jp)

Figure 1A

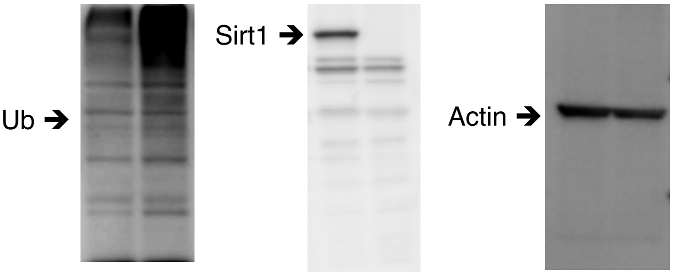

Figure 1B

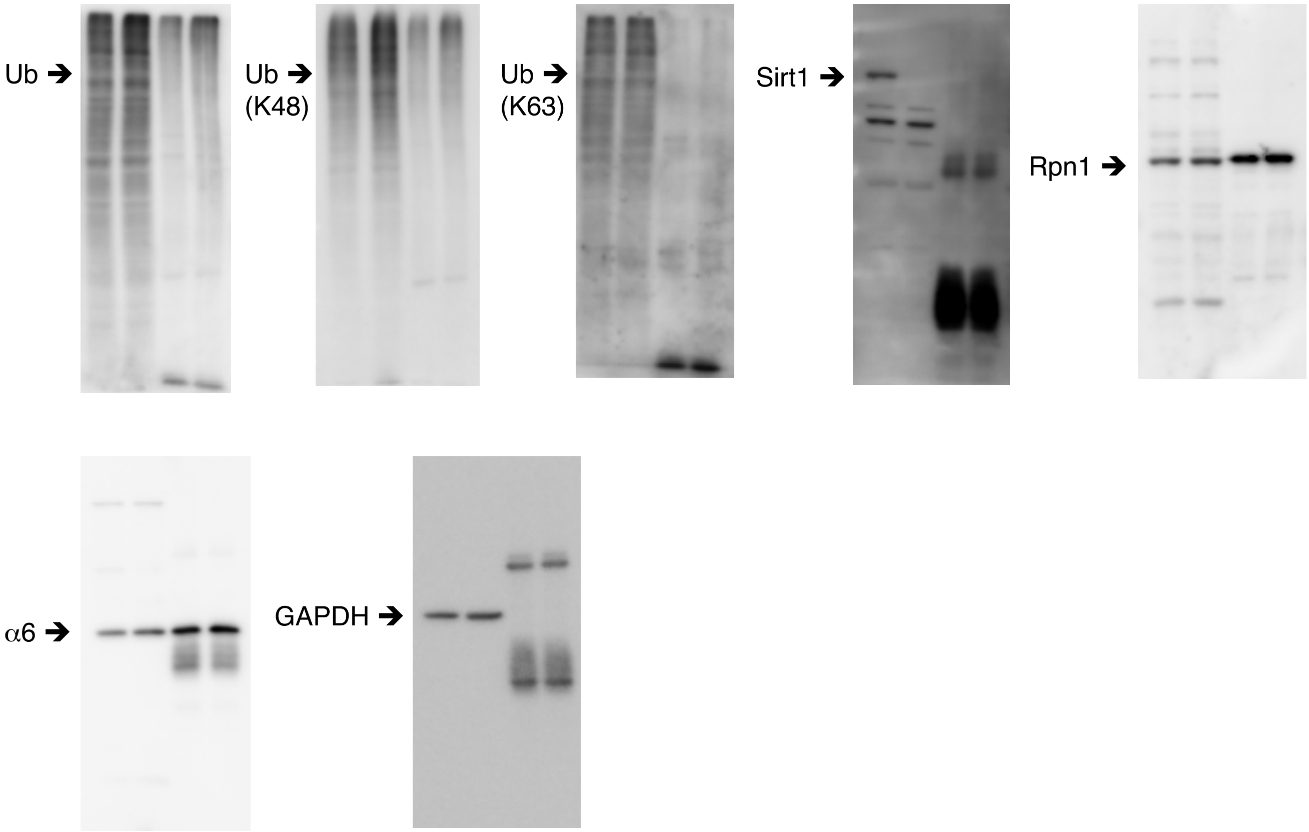

Figure 1C

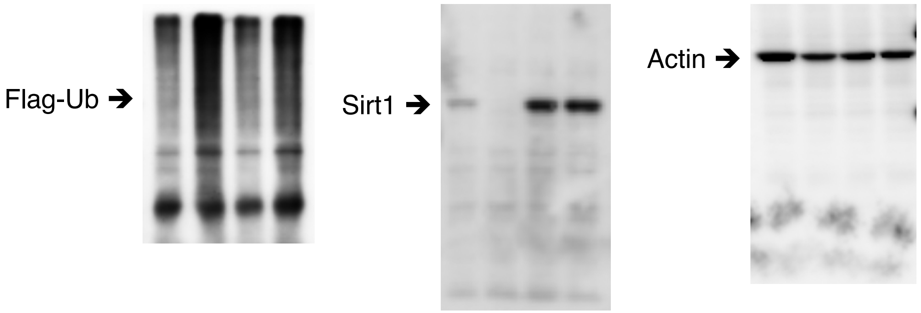

Figure 2A

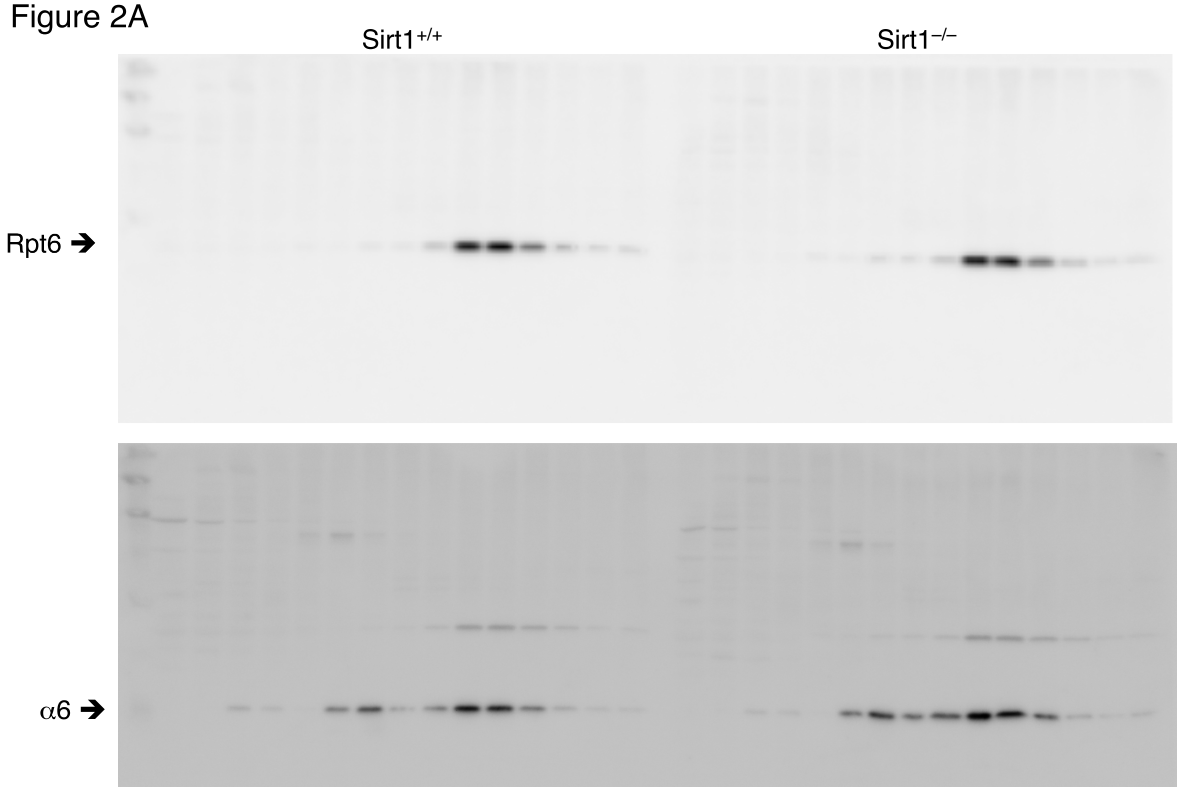

Figure 2C

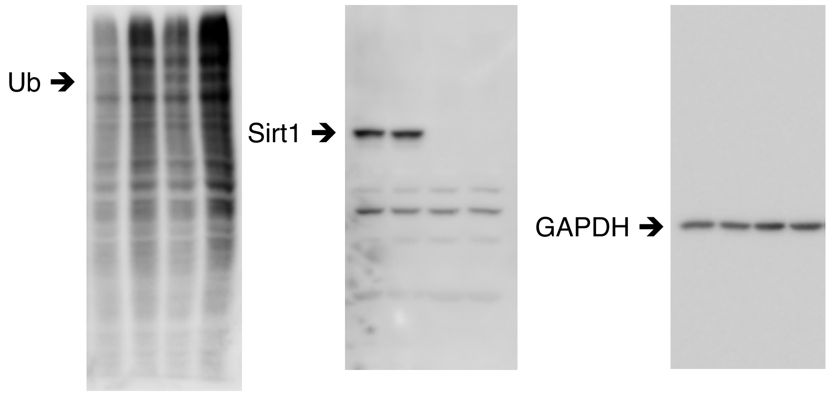

Figure 2D

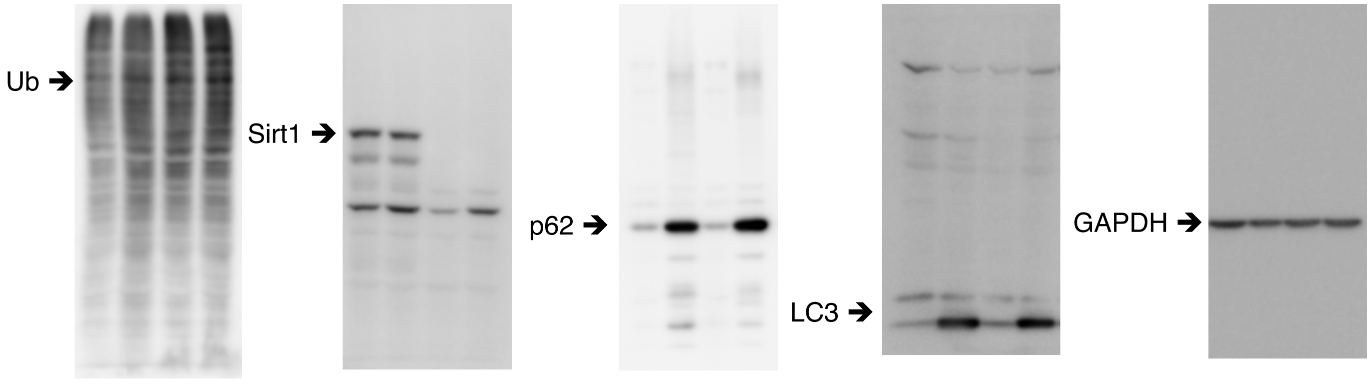

Figure 3A

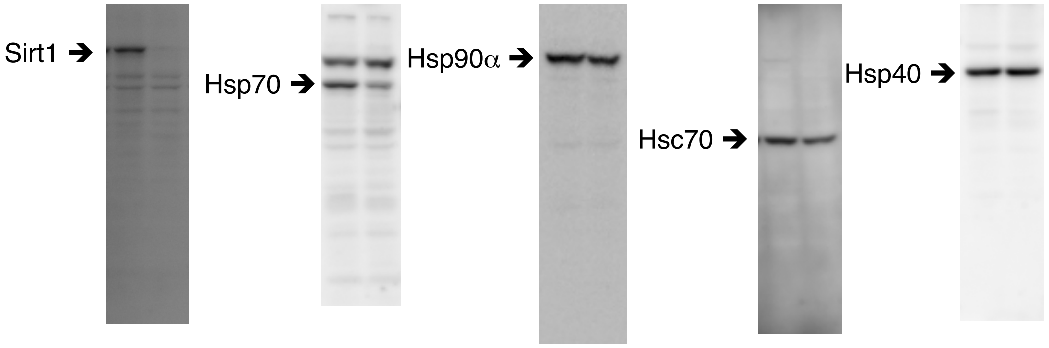

Figure 3B

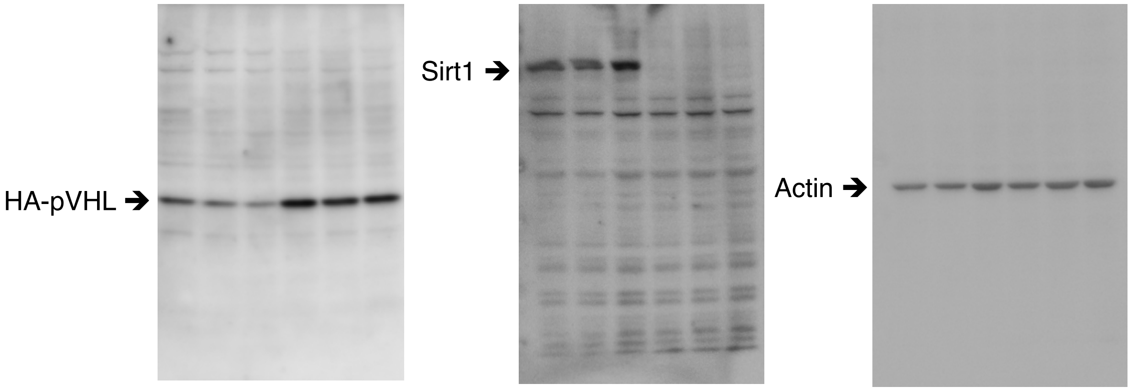

Figure 4A

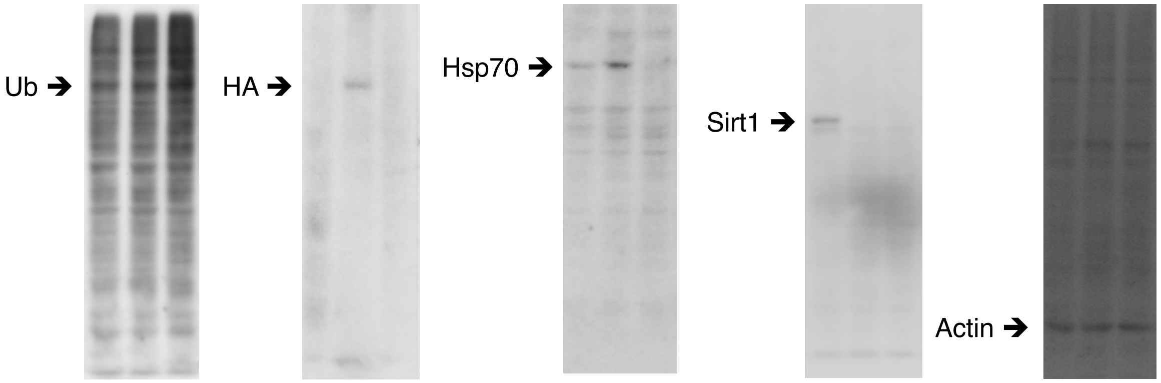

Figure 4B

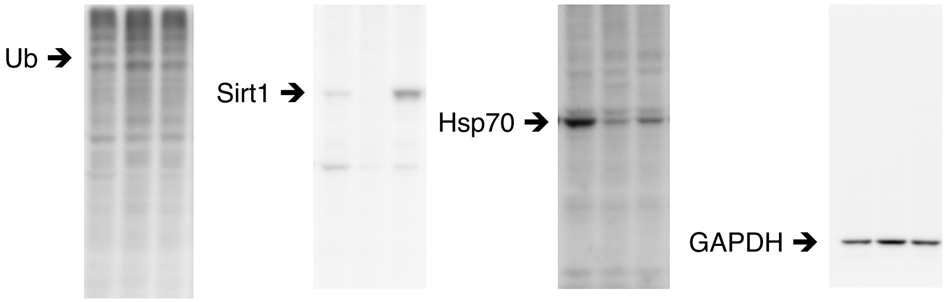

Figure 5B

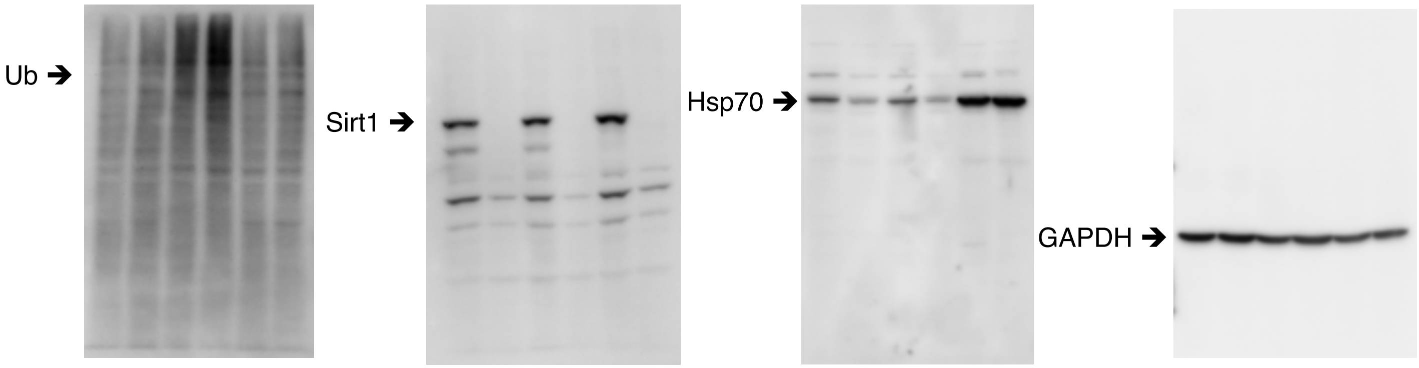

Figure 5C

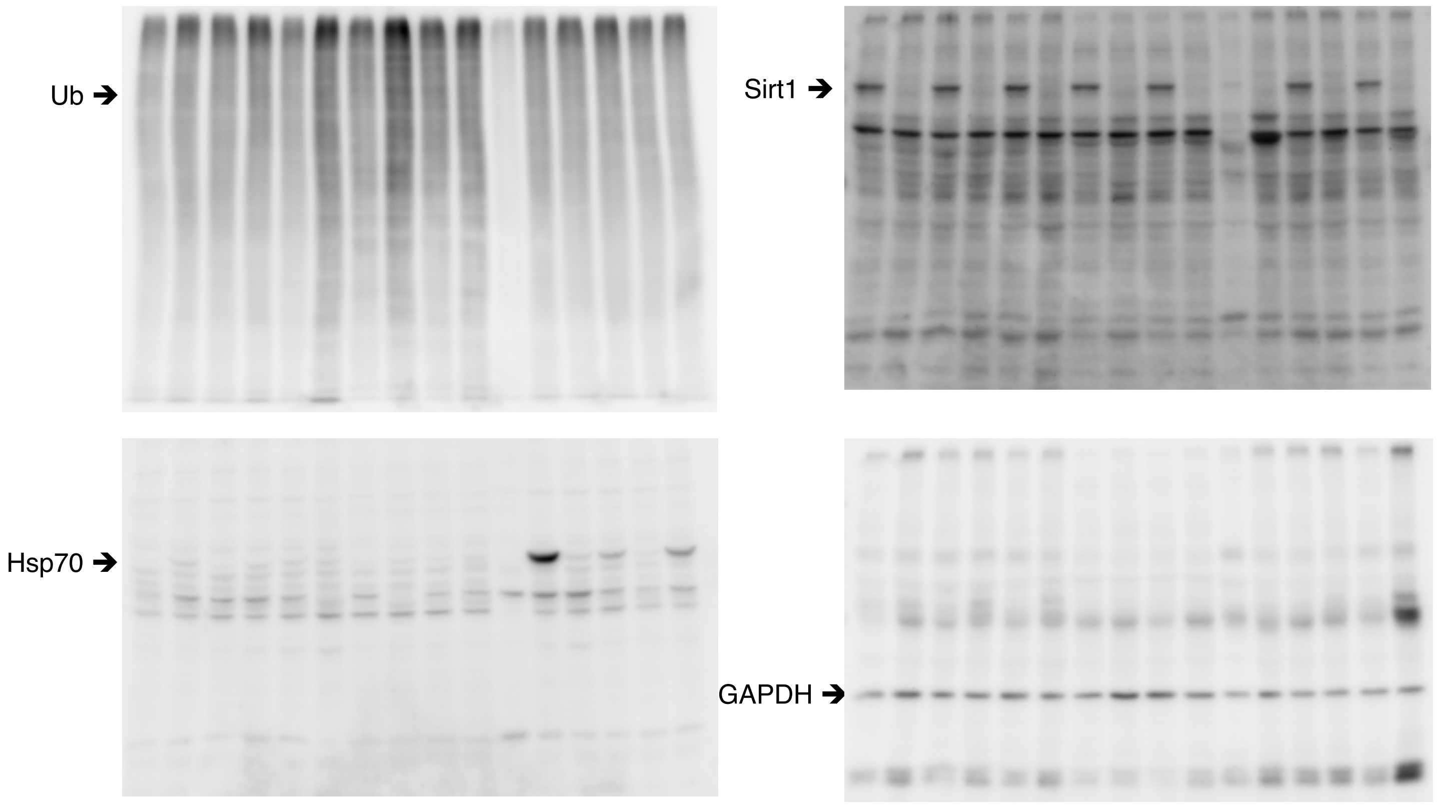

Figure 5D

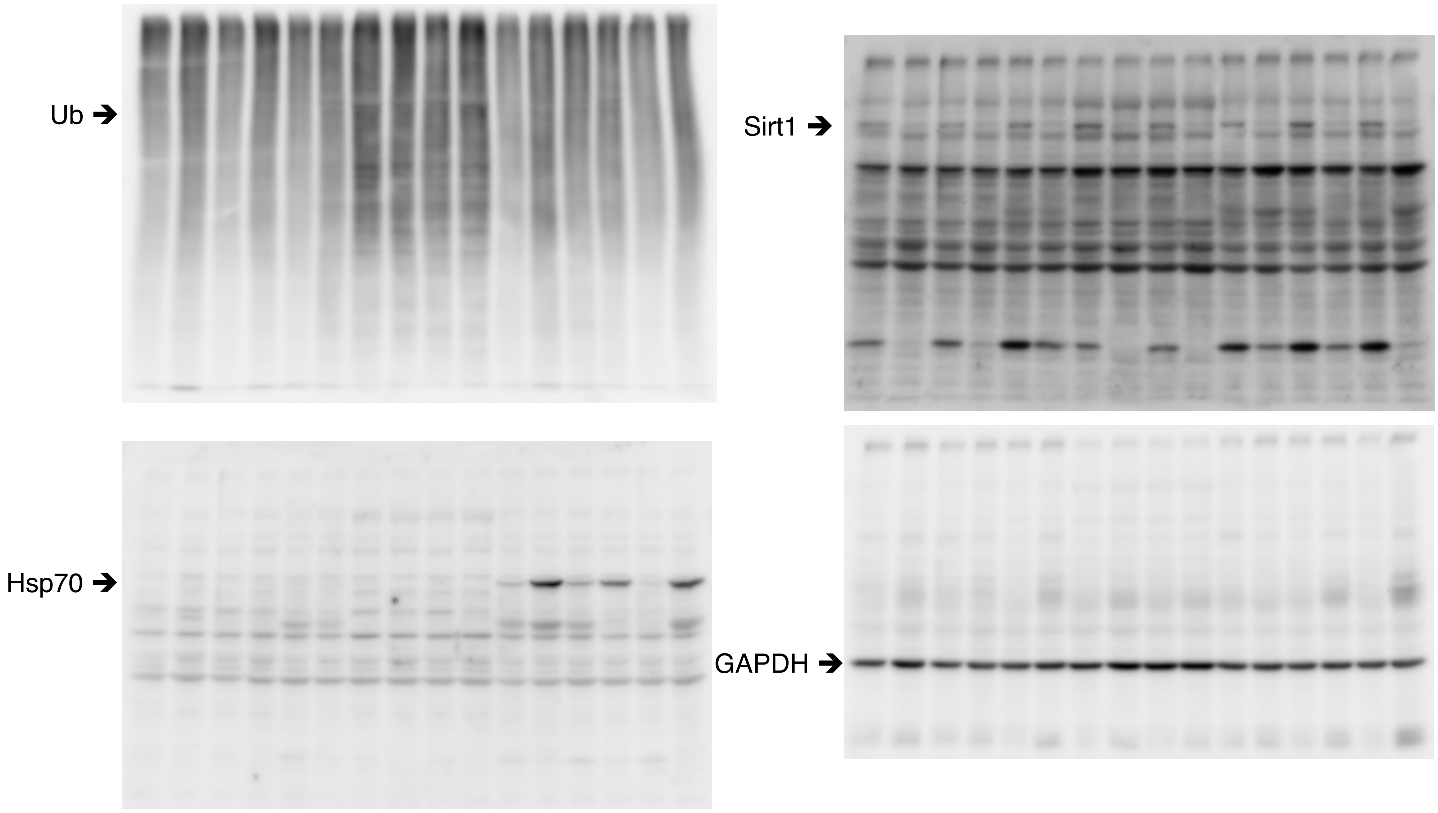

Supplementary Figure

Uncropped gel images in this study.
